# Assessing genotype adaptability and stability in perennial forage breeding trials using random regression models for longitudinal dry matter yield data

**DOI:** 10.1093/g3journal/jkae306

**Published:** 2025-02-14

**Authors:** Claudio Carlos Fernandes Filho, Sanzio Carvalho Lima Barrios, Mateus Figueiredo Santos, Jose Airton Rodrigues Nunes, Cacilda Borges do Valle, Liana Jank, Esteban Fernando Rios

**Affiliations:** Sugarcane Technology Center, Piracicaba, SP 13400-970, Brazil; Embrapa Beef Cattle, Campo Grande, MS 79106-550, Brazil; Embrapa Beef Cattle, Campo Grande, MS 79106-550, Brazil; Department of Biology, Institute of Natural Sciences, Universidade Federal de Lavras, Minas Gerais 37200-900, Brazil; Embrapa Beef Cattle, Campo Grande, MS 79106-550, Brazil; Embrapa Beef Cattle, Campo Grande, MS 79106-550, Brazil; Agronomy Department, University of Florida, Gainesville, FL 32611-0180, USA

**Keywords:** random regression, genotype by environment, longitudinal data, forage breeding

## Abstract

Genotype selection for dry matter yield (DMY) in perennial forage species is based on repeated measurements over time, referred to as longitudinal data. These datasets capture temporal trends and variability, which are critical for identifying genotypes with desirable performance across seasons. In this study, we have presented a random regression model (RRM) approach for selecting genotypes based on longitudinal DMY data generated from 10 breeding trials and three perennial species, alfalfa (*Medicago sativa* L.), guineagrass (*Megathyrsus maximus*), and brachiaria (*Urochloa spp.*). We also proposed the estimation of adaptability based on the area under the curve and stability based on the curve coefficient of variation. Our results showed that RRM always approximated the (co)variance structure into an autoregressive pattern. Furthermore, RRM can offer useful information about longitudinal data in forage breeding trials, where the breeder can select genotypes based on their seasonality by interpreting reaction norms. Therefore, we recommend using RRM for longitudinal traits in breeding trials for perennial species.

## Introduction

Genotype selection in perennial forage species involves multiple assessments conducted repeatedly over time, across various seasons and years. Therefore, the evaluation of multiharvest forage breeding trials is time-consuming and expensive. In this context, proper statistical methods that accurately predict the true genotypic values are crucial (Smith and Spangenberg 2014). As the genotypes experience different environmental conditions over time, it is expected that differential gene expression occurring throughout the growing season. Therefore, in a multiharvest trial, a response variable can be treated as different traits in a multivariate framework analysis (Falconer and Mackay 1996). In this way, the genetic correlation between the same traits in different harvests is a measure of genotype by harvest interaction (G × H) (Apiolaza and Garrick 2001; Crossa et al. 2004; Van Eeuwijk et al. 2016). Multiharvest trials can also be described as a special case of multienvironment trials, in which the environments represent the different time points when data are collected in the same trial. The repeated measurement of the same trait over time generates longitudinal datasets and the sequential nature of measurements creates patterns of variation (Hand and Crowder 2017).

There are several models to deal with longitudinal datasets and the most common and simple (co)variance structure is the first-order autoregressive (AR1), where a single correlation parameter (*ρ*) is estimated. The model postulates a mechanism where the correlation between measurements *j* and *k* is $\rho^{\left|\;j-k\right|}$, where the genotypic value of the genotype is a function of genes acting in a given time plus genes acting on the new measurement (Apiolaza and Garrick 2001). The AR1 model is an appealing method for modeling (co)variance structure for genotypes measured over time (Apiolaza and Garrick 2001; Yang et al. 2006; Vanhatalo et al. 2019). However, the AR1 model is recommended when the period between measurements is equally spaced, and when time points are unequally spaced nonlinear restrictions should be imposed for parameter estimation (Mckenzie 2001). Due to the yield seasonality irregular time series are frequently observed in perennial forage yield measurements. The yield seasonality in forages is characterized by variation in growth and quality in response to environmental conditions (Reis and Rosa 2001). Therefore, plants grow faster under favorable climate conditions and harvests are more frequent, whereas harvests are less frequent under unfavorable conditions. Thus, the time series for forage yield measures are naturally irregular.

Random regression models (RRMs) were introduced by Henderson (1982) and Laird and Ware (1982). Schaeffer and Dekkers (1994) suggested their use in dairy cattle (*Bos taurus*) breeding to analyze day production records. Since then, several studies used RRM to predict growth in sheep (*Ovis aries*) (Lewis and Brotherstone 2002), body weight in beef cattle (Arango et al. 2004), body weight in swine (*Sus scrofa domesticus*) (Huisman et al. 2002), and egg production in layer (*Gallus gallus domesticus*) (Wolc et al. 2011). Recently, RRM was applied to longitudinal data from perennial forage breeding trials for dry mater yield (DMY) in elephant grass (*Pennisetum purpureum* Schmach.) (Rocha et al. 2018) and for forage quality in ryegrass (*Lolium perenne L*.) (Bornhofen et al. 2022). The use of RRM has also been increasing for annual crops with the advent of high-throughput phenotyping, which generates longitudinal datasets (Sun et al. 2017; Campbell et al. 2018; Moreira et al. 2021). RRM can deal with longitudinal data (Schaeffer 2004) as it captures the change of a trait continuously over time with few parameters by covariance functions (e.g. orthogonal polynomials and splines) (Kirkpatrick et al. 1990; Meyer 1998). Kirkpatrick et al. (1990) reported that RRM deals with unequally time-spaced measurements, relating that RRM should be the adequate model under this condition. Furthermore, there is a possibility to include environmental-dependent covariate in RRM, e.g. temperature and humidity to study the genotypes' response to abiotic stress (Brügemann et al. 2011; Bohlouli et al. 2013; Mbuthia et al. 2021).

In this study, we investigated the use of RRM for longitudinal data of dry matter yield in ten forage breeding trials for three different species (alfalfa, *M. maximus* and *Urochloa spp.)* for genotype selection and genetic interpretation.

## Material and methods

### Datasets

We used data from three forage species in 10 trials (T1 to T10) conducted from 2015 to 2020 in three locations and following different experimental designs (augmented row column design, ARCD; alpha-lattice design, ALD; randomized complete block design, RCBD) (Table 1). Total dry matter yield (DMY, kg.ha^−1^) was assessed in all trials. The number of genotypes evaluated varied from eight (T4 and T5) to 182 (T1). The dataset is composed of data from early breeding trials (T1, T2, T3, T8, and T10) in which many genotypes are tested, and advanced breeding trials with a lower number of genotypes (T4, T5, T6, T7, and T9) (Table 1). The number of harvests in each trial varied from six (T7, T8, and T10) to 16 (T4 and T5).

**Table 1.** Description of experimental layout for the three forage species evaluated from 2015 to 2020.

Trial	Species	Year	Location	Design	Genotypes	Harvests	Columns	Rows	Plots
T1	Alfalfa	2018–2019	Citra—FL—USA	ARCD	182	11	32	14	405
T2	M. maximus	2016–2019	Campo Grande—MS—BR	ALD	110	9	22	20	330
T3	M. maximus	2016–2019	Campo Grande—MS—BR	ALD	110	9	22	20	330
T4	*U. brizantha*	2009–2011	Campo Grande—MS—BR	RCBD	8	16	—	—	32
T5	*U. brizantha*	2009–2011	Terenos—MS—BR	RCBD	8	16	—	—	32
T6	*U. decumbens*	2018–2019	Campo Grande—MS—BR	RCBD	9	13	9	4	36
T7	*U. decumbens*	2018–2019	Brasilia—DF—BR	RCBD	12	6	12	4	48
T8	*U.* (Inter)	2015–2016	Campo Grande—MS—BR	RCBD	99	6	8	50	396
T9	*U.* (Inter)	2019–2020	Campo Grande—MS—BR	RCBD	15	10	6	11	60
T10	*U. decumbens*	2015–2016	Campo Grande—MS—BR	RCBD	36	6	3	50	144

### Statistical analysis

Analyzes for each multiharvest trial (same trial over time) were performed based on a two-stage analysis using the weighting method proposed by Smith et al. (2001). In a two-stage analysis, genotypes' best linear unbiased estimates (BLUEs) from each harvest in stage one were combined in a weighted multiharvest mixed model analysis in stage two, where the weights provide a measure of relative uncertainty of the estimated genotypes' BLUES from each harvest (Smith et al. 2001; Möhring and Piepho 2009; Gogel et al. 2018). All the analyses were done by using ASREML-R (Butler et al. 2017) and SpATS (Rodríguez-Álvarez et al. 2018) R packages, and the data summarization through graphs was done by ggplot2 (Wickham et al. 2016) R package. The scripts for the analysis can be found at github (https://github.com/claudiocff/RRM-and-FAMM-asreml-two-step).

#### First stage: estimating BLUEs and weights accounting for spatial variation

We obtained the BLUEs and weights of the genotypes at each harvest by trial, using the SpATS R package (Rodríguez-Álvarez et al. 2018) in a mixed model framework:


(1) $$y=X\beta +X_{s}\beta_{s}+Z_{s}u_{s}+Z_{c}u_{c}+Z_{r}u_{r}+e$$

(2) $$y=X\beta +X_{s}\beta_{s}+Z_{s}u_{s}+Z_{b}u_{b}+e$$

where *y* is the vector from measured DMY from each plot; *β* is the vector of the fixed effects of genotypes and replication; $u_{r}$ and $u_{c}$ are the vectors of random effects of rows and columns, respectively in the augmented row column design (T1—Table 1), where $u_{r}∼N(0,I_{r}\sigma_{r}^{2})$ and $u_{c}∼N(0,I_{c}\sigma_{c}^{2})$; $u_{b}$ is the vector of random effects of block effects in a randomized complete block design (T4, T5, T6, T7, T8, T9, and T10—Table 1), or the vector of the random effects of incomplete blocks within replication in the alpha-lattice design (T2 and T3—Table 1), in which $u_{b}∼N(0,I_{b}\sigma_{b}^{2})$; $\beta_{s}$ is the vector of fixed effects of the smooth spatial surface (unpenalized); $u_{s}$ is the vector of random effects of the penalized part of the smooth surface (penalized). The fixed term ($X_{s}\beta_{s}$, unpenalized) and random term ($Z_{s}u_{s}$, penalized) describe the mixed model expression of the smooth spatial surface ($f(r,c)=X_{s}\beta_{s}+Z_{s}u_{s}$), where the random spatial vector $u_{s}$ has (co)variance matrix *S.* The SpATS model uses the P-spline ANOVA (PS-ANOVA) to describe the 2D-splines in the mixed model framework. The $X_{s}$, $Z_{s}$ incidence matrices, and the (co)variance matrix *S* are described by Lee et al. (2013) and Rodríguez-Álvarez et al. (2018). The PS-ANOVA parametrization can be decomposed as a linear sum of the univariate and bivariate smooth functions (Velazco et al. 2017); *e* is the vector of random errors, $e∼N(0,I_{e}\sigma_{e}^{2})$; $\sigma_{r}^{2}$, $\sigma_{c}^{2}$, $\sigma_{b}^{2}$, and $\sigma_{e}^{2}$ are the variance components associated with the random effects of rows, columns, blocks, and errors. *X*, $Z_{r}$, $Z_{c}$, and $Z_{b}$ are incidence matrices for the fixed effects, the random effects of rows, columns and blocks, respectively. $I_{r}$, $I_{c}$, $I_{b}$, and $I_{e}$ are identity matrices.

#### Second stage: modeling genotype by harvest interaction

For the statistical model described below, the BLUEs obtained for each genotype in each harvest by trial were regressed on a time gradient (days), where the first harvest of each trial were considered as day zero and the other harvest times were the days after the first harvest. Therefore, random coefficients were computed for each genotype to describe the “genotypes” DMY trajectory over time. Polynomial functions were used to model the longitudinal dimensions by using orthogonal Legendre polynomials (Kirkpatrick et al. 1990). The orthogonal polynomials were obtained by rescaling the time points from −1 to 1 using the expression:


(3) $$t_{i}=-1+2\left(\frac{t_{i}-t_{\min}}{t_{\max}-t_{\min}}\right)$$

the Legendre polynomials are denoted by $P_{n}(t)$. Defining $P_{0}(t)=1$, the polynomial *n + 1* is described by the recursive equation:


(4) $$P_{n+1}(t)=\frac{1}{n+1}[\left(2n+1)tP_{n}(t)-nP_{n-1}(t\right)]$$

on the normalized form the Legendre polynomial can be described:


(5) $$\varphi_{n}(t)={\left(\frac{2n+1}{2}\right)}^{0.5}P_{n}(t)$$

therefore, for a polynomial of order two we can obtain the following equations:


$$\varphi_{0}(t)={\left(\frac{1}{2}\right)}^{0.5}P_{0}(t)=0.7071\varphi_{1}(t)={\left(\frac{3}{2}\right)}^{0.5}P_{1}(t)=1.22467t$$

(6) $$\varphi_{2}(t)={\left(\frac{5}{2}\right)}^{0.5}\left(\frac{3}{2}t^{2}-\frac{1}{2}\right)=-0.7906+2.3717t^{2}$$

considering *m = 2* as the order of the covariance function to be used, the Legendre coefficient matrix *Λ* will have dimensions of (m+1)×(m+1) can be defined as:


(7) $$\Lambda =\left[\begin{array}{ccc} 0.7071 & 0 & -0.7906\\ 0 & 1.2247 & 0\\ 0 & 0 & 2.3717 \end{array}\right]\;$$

considering four different time points and an order two polynomial, the incidence matrix of time points (*M*) can be defined as:


(8) $$M=\left[\begin{array}{c} \begin{array}{ccc} 1 & t_{0} & t_{0}^{2} \end{array}\\ \begin{array}{ccc} 1 & t_{1} & t_{1}^{2} \end{array}\\ \begin{array}{ccc} 1 & t_{2} & t_{2}^{2} \end{array}\\ \begin{array}{ccc} 1 & t_{3} & t_{3}^{2} \end{array} \end{array}\right]\;$$

where *t_i_* is the time point scaled by equation (3).

Finally, the Legendre polynomials can be computed as Φ=MΛ, where Φ is a matrix containing the normalized polynomials for harvest time; *M* store polynomials of standardized harvest times; *Λ* is the matrix of Legendre polynomial coefficients of order *m + 1*, where *m* is the degree of fit (Schaeffer 2016). The random regression model can be defined as:


(9) $$y=\Phi_{1}\beta +\Phi_{2}u_{g}+e$$

where *y* is the vector of BLUEs estimated by the models (1) or (2); *β* is the vector of the fixed regression coefficients; $\;u_{g}$ is the vector of random regression coefficients of genotypes, in which $u_{g}∼N$$\left(0,K_{g}⊗I_{g}\sigma_{g}^{2}\right)$; *e* is the vector of the errors, where $e∼N(0,R\sigma_{e}^{2})$. *K_g_* is an unstructured (co)variance matrix associated to the random regression coefficients; the matrix *K_g_* can be described as:


(10) $$K_{g}=\left[\begin{array}{cccc} \sigma_{g_{0}}^{2} & \sigma_{g_{0}g_{1}} & \cdots & \sigma_{g_{0}g_{m}}\\ & \sigma_{g_{1}}^{2} & \cdots & \sigma_{g_{1}g_{m}}\\ & & \ddots & \vdots \\ Sym. & & & \sigma_{g_{m}}^{2} \end{array}\right]\;$$

where, $\sigma_{g_{m}}^{2}$ is the variance component associated to the coefficient of order *m*; $\sigma_{g_{n}g_{m}}$ is the covariance between the coefficient of order *m* and *n.*

*R* is the variance matrix of the errors, where $R=\mathrm{diag}(R_{1}\cdot \cdot \cdot R_{j})$, in which *j* is the harvest by trial. In practice, $R_{j}$ are unknown and replaced by an estimate ${\hat{R}}_{j}$ from each harvest. It is sometimes not feasible to store and use the full matrix ${\hat{R}}_{j}$ from each harvest, and so a vector of approximate weights is required. We used the weights proposed by Smith et al. (2001), where the weights are based on the diagonal elements of ${\hat{R}}_{j}^{-1}$ designated as *Π*, in which:


(11) $$\Pi =\mathrm{diag}(\pi_{1}^{T}\cdot \cdot \cdot \pi_{j}^{T}),$$

where $\pi_{j}$ consists of the diagonal elements of ${\hat{R}}_{j}^{-1}$. This simple approximation reflects the uncertainty in each estimated BLUE, accounting for within-trial heterogeneity, differing replication and spatial trends.

Based on Kirkpatrick et al. (1990), the following estimator was used to obtain the genetic variance and covariance components across harvest times (${\hat{\Sigma }}_{g}$) on original scale:


(12) $${\hat{\Sigma }}_{g}=\Phi_{2}K_{g}\Phi_{2}^{T},$$

where $\Phi_{2}$ is the incidence matrix of the Legendre polynomials associated to the random effects of genotypes; *K_g_* is the (co)variance matrix associated to the random genotypes' coefficients, defined in (10).

The genotypic values for each genotype across harvest time can be estimated by the equation:


(13) $$G_{V}=J\beta^{T}\Phi_{1}^{T}+U_{g}\Phi_{2}^{T},$$

where $G_{V}$ is a *i × j* matrix of the genotypic values on the original scale, where *i* is the number of genotypes and *j* the number of harvest time points; *J* is a column vector of 1's size equal the number of genotypes (*i*); $\beta^{T}$ is the transposed vector of the fixed regression coefficients of size 1 *×* (*d + 1*), in which *d* is the degree of the polynomial fitted for the fixed regression; $\Phi_{1}^{T}$ is the transposed incidence matrix of the Legendre polynomials for each harvest time for the fixed regression with size (*d + 1) × j*; $U_{g}$ is the genotypes's random coefficients matrix, size *i ×* (*m* + 1); $\Phi_{2}^{T}$ is the transposed incidence matrix of the Legendre polynomials for each harvest time for the random regression with size (*d + 1) × j.*

**Fig. 1.**
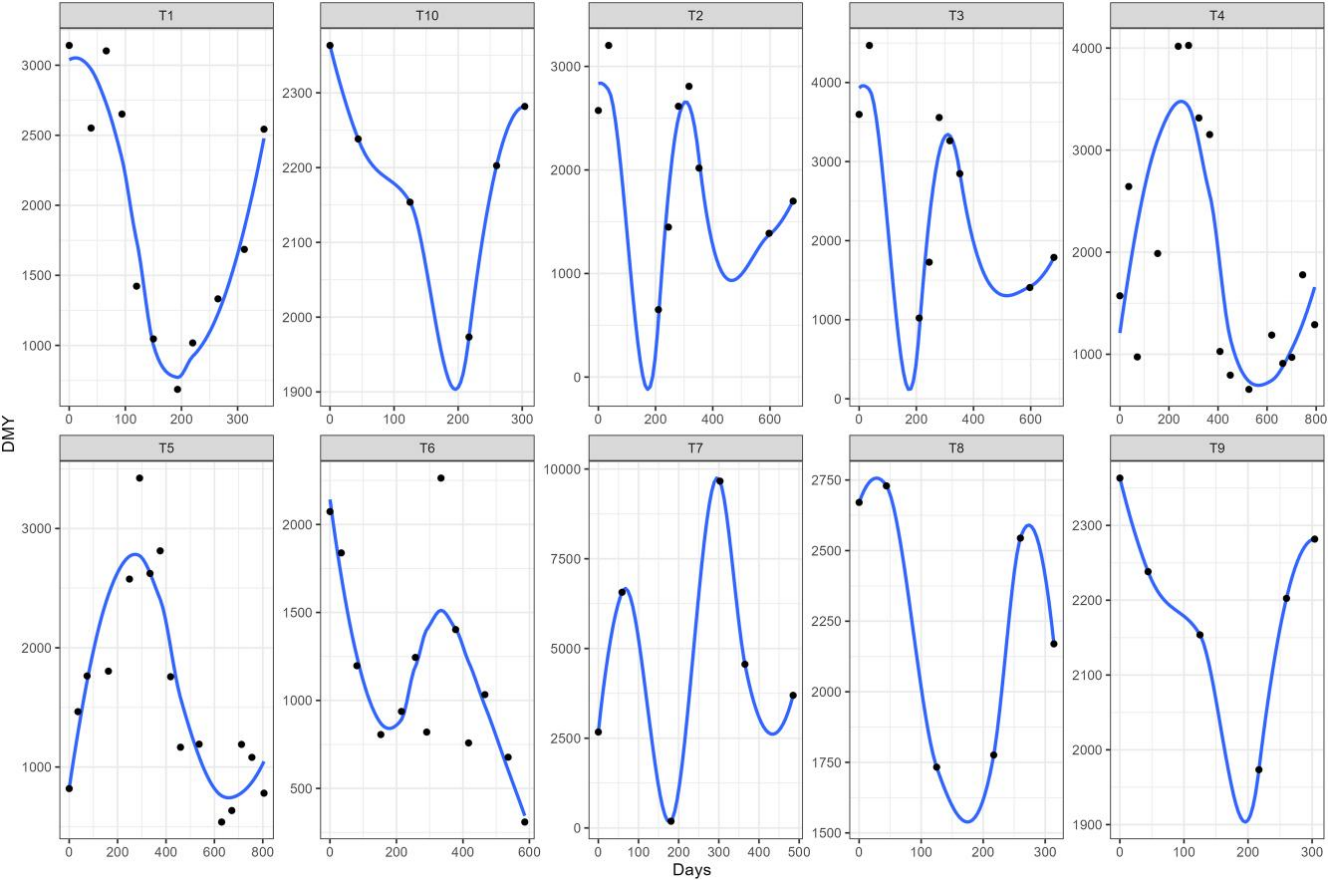
Mean dry matter yield (kg.ha^−1^) trajectory over time for trials T1 to T10. The dots represent the observed means over time.

The polynomial function for the fixed regression was defined graphically by using a loess function, where the function order was determined by the number of curves (*c*) + 1 in the mean DMY trajectory across harvest time (Fig. 1). For example, for the trials T1 and T5 a polynomial of degree three were fitted, since two curves were observed on the mean DMY trajectory. The random polynomial regression degree was determined by the Bayesian information criteria (BIC).

#### Heritability, broad adaptability, and stability

The heritability over harvest times was estimated by the expression:


(14) $$H_{j}^{2}=\frac{\mathrm{diag}(\Sigma_{g})}{\mathrm{diag}(\Sigma_{g})+\frac{{\bar{\sigma }}_{e}^{2}}{r}}$$

where $H_{j}^{2}$ is the genotype mean-based heritability estimated at each harvest time; $\Sigma_{g}$ is the genetic variance–covariance matrix estimated by equation (12); ${\bar{\sigma }}_{e}^{2}$ is the mean error variance component across harvests; *r* is the number of replications in the trial.

The broad adaptability for each genotype was estimated based on the area under the DMY trajectory curve, in which reflects the total DMY accumulation over harvest time:


(15) $$\begin{array}{r} A_{i}=\overset{1}{\underset{-1}{\int }}\;\underset{\mathrm{Fixed}}{\underbrace{b_{0}+b_{1}t+b_{2}t^{2}+\ldots +b_{d}t^{d}}}+\underset{\mathrm{Random}}{\underbrace{g_{i0}+g_{i1}t+g_{i2}t^{2}+\ldots +g_{im}t^{m}}}d(t) \end{array}$$

where *A_i_* is the area under the trajectory curve of the genotype *i*; *b_0_* is the fixed regression intercept; *g_i0_* is the random regression intercept of the genotype *i*; *t* is the harvest time point; *d* and *m* are the polynomial fitted degree for the fixed and random regression, respectively; *b_d_* is the fixed regression coefficient of degree *d;*  $g_{im}$ is the random regression coefficient of degree *m* for genotype *i.*

The genotypes' stability was calculated based on the trajectory curve's coefficient of variation (*CV_c_*), in which reflect the genotypes' Type I stability, where the genotype is stable if present small variance between environments (harvests), also called biological stability (Lin et al. 1986):


(16) $$CV_{c_{i}}=\frac{\sigma_{c_{i}}}{b_{0}+g_{i0}}$$

where $\sigma_{c_{i}}$ is the standard trajectory curve deviation for genotype *i*; $b_{0}+g_{i0}$ is the overall performance for genotype *i.*

#####  

###### Genetic interpretation on random regression models

One of the advantages of random regression models is the use of eigenfunction $\left(\Psi_{k}\right)$ of the genetic coefficient (co)variance matrix (10), in which can provide genetic insights about the studied trait based on Kirkpatrick et al. 1990:


(17) $$\Psi_{k}=\overset{M}{\underset{m=0}{\sum }}\;(v_{\Psi k}{)}_{m}\Phi_{m}$$

where $(v_{\Psi k}{)}_{m}$ is the *m*th element of the *k*th eigenvector of *K_g_*, and $\Phi_{m}$ is the normalized value of the *m*th Legendre polynomial.

## Results

### Overall description of RRM across trials

The degree of the polynomial used to fit the fixed part of the RRM varied from 2 (in T10) to 5 (in T2 and T3) (Table 2). However, there was no clear trend between the number of harvests evaluated in each trial and the order of the polynomial used (Tables 1  2). The choice of polynomial degree for the fixed part of the model was based on the number of contrasting seasons evaluated in each trial. These contrasting seasons resulted in pits and peaks that had to be modeled by the fixed polynomial. On the other hand, for the random part of the RRM, lower-order polynomials were mostly preferred. In most trials, a first-order polynomial was sufficient to model the genotype by harvest interaction (G × H). A second-order polynomial was used in trials T4 and T8, and a third-order polynomial was employed in trial T1 (Table 2).

**Table 2.** Genetic parameters estimated using random regression models for alfalfa (T1), *M. maximus* (T2 and T3), and *Urochloa spp*. (T4 to T10).

Trial	Degree (F)	Degree (R)	*H^2^*	$\rho_{g}$
T1	3	3	0.36 (0.11)	0.78 (0.19)
T2	5	1	0.63 (0.02)	0.98 (0.02)
T3	5	1	0.51 (0.01)	0.90 (0.10)
T4	3	2	0.34 (0.20)	0.26 (0.54)
T5	3	1	0.52 (0.10)	0.67 (0.33)
T6	3	1	0.16 (0.08)	0.75 (0.28)
T7	4	1	0.42 (0.18)	0.29 (0.61)
T8	4	2	0.69 (0.02)	0.82 (0.18)
T9	3	1	0.31 (0.03)	0.89 (0.11)
T10	2	1	0.55 (0.07)	0.88 (0.11)

Fitted polynomial order for fixed (F) and random (R) regression, mean heritability (*H^2^*), and genetic correlation between harvests $\left(\rho_{g}\right)$ for each trial estimated by RRM. The values between parenthesis represent the standard deviations across harvests for *H^2^* and among pair of harvests in case of $\rho_{g}$.

Heritability estimates varied across harvests, ranging from low (0.16 in T6) to moderate (0.69 in T8), as presented in Table 2. Generally, heritability showed limited variation across harvests within most trials, and high genetic correlations were observed between harvests. This pattern suggests a predominance of noncrossover genotype-by-harvest interactions, where genotype rankings remain relatively stable. Exceptions to this trend were noted in trials T4 and T7, which exhibited lower genetic correlations, indicative of more complex genotype-by-harvest interactions.

**Fig. 2.**
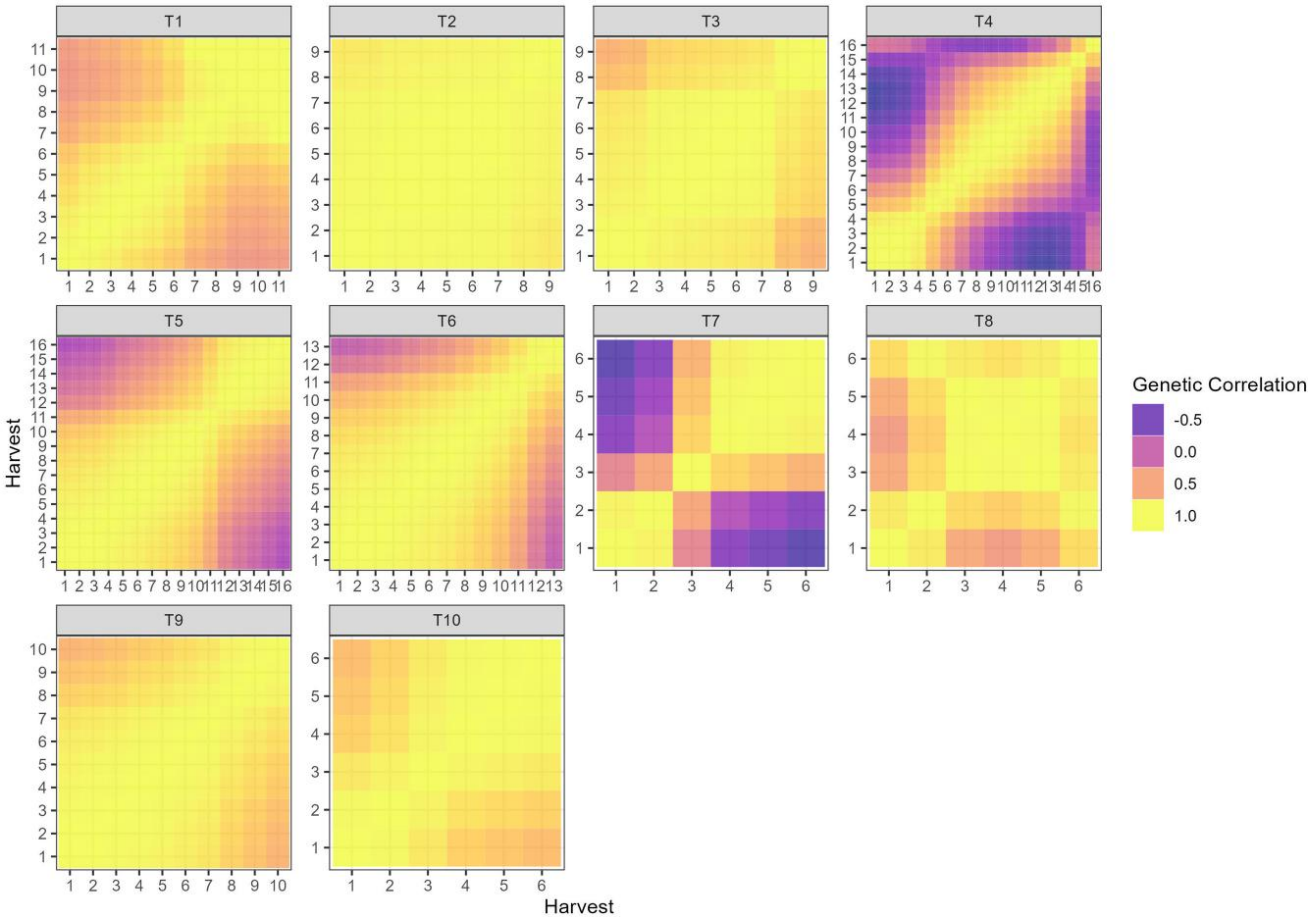
Heatmap of the genetic correlations between harvests estimated by random regression models alfalfa (T1), *M. maximus* (T2 and T3), and *Urochloa spp* (T4 to T10).

The RRM models estimated an autoregressive covariance pattern for all datasets, indicating that correlations were higher between harvests that were closer in time and decreased as the time gap between harvests increased (Fig. 2). This pattern reflects the expected temporal genetic relationship in longitudinal data. However, trials T4, T5, T6, and T7 demonstrated a notable exception, with higher levels of crossover interactions, evidenced by the presence of negative correlations between some harvests (Fig. 2). These negative correlations suggest significant changes in genotype performance over time, which may be attributed to the small number of genotypes evaluated in these trials (Table 1), potentially increasing the variability in genotype-by-harvest interactions.

To provide a deeper interpretation of the RRM models for genotype selection, we focused on trials with a higher level of complexity in genotype-by-environment interactions, characterized by low to moderate genetic correlations between harvests. For this purpose, trials T1, T5, and T7 were selected for further analysis and discussion due to their diverse correlation structures and ability to capture these interactions effectively (Table 2 and Fig. 2). This selection allows us to highlight the implications of genotype-by-harvest interactions for breeding decisions, especially under varying environmental and temporal conditions.

### Genotype selection and G × H interpretation

#### Alfalfa breeding trial—T1

In this trial, 182 genotypes were evaluated over 11 harvests. To fit the model G × H for this trial, we used a Legendre polynomial of degree three for both the fixed and random effects (Table 2), which resulted in the estimation of 12 parameters.

#### Variance components, heritability, and genetic behavior

The polynomial genetic variances varied from 2,930 to 343,654 for *g_3_* and *g_0_*, respectively. The genotypes' intercept (*g_0_*) retained most of the genetic variance, explaining 74% of the genetic variance, and the components related to the genotype's curve shape (*g_1_*, *g_2,_* and *g_3_*) accounted to 26% of the genetic variance (Table 3). The most important correlation between the polynomial coefficients and the easiest to interpret is the correlation between *g_0_* and *g_1_*, it shows the genetic variance behavior over time. In this trial, the correlation between *g_0_* and *g_1_* was negative (−0.22, Table 3), indicating lower genetic variance can be observed over time.

**Table 3.** Summary of genetic and nongenetic parameters estimated by RRM for alfalfa trial (T1).

	$g_{0}$	$g_{1}$	$g_{2}$	$g_{3}$
$g_{0}$	343,654	−0.22	0.76	0.03
$g_{1}$	—	101,118	−0.61	−0.07
$g_{2}$	—	—	19,627	−0.63
$g_{3}$	—	—	—	2930
Importance (%)	74	21	4	1
${\bar{\sigma }}_{e}^{2}$	365,436			

$g_{0}$ , $g_{1}$, $g_{2}$, and $g_{3}$ are the random regression intercept and the first-, second-, and third-order coefficient, respectively. ${\bar{\sigma }}_{e}^{2}$ is the mean error variance across harvests. The diagonal elements of the table are the genetic variances associated with the intercept and polynomial coefficients ($\sigma_{g_{0}}^{2},\sigma_{g_{1}}^{2},\sigma_{g_{2}}^{2},\sigma_{g_{3}}^{2}$); the off diagonal of the table are the correlations between intercept and polynomial coefficients ($\rho_{g_{0}g_{1}},\rho_{g_{0}g_{2}},\rho_{g_{0}g_{3}},\rho_{g_{1}g_{2}},\rho_{g_{1}g_{3}},\rho_{g_{2}g_{3}}$).

**Fig. 3.**
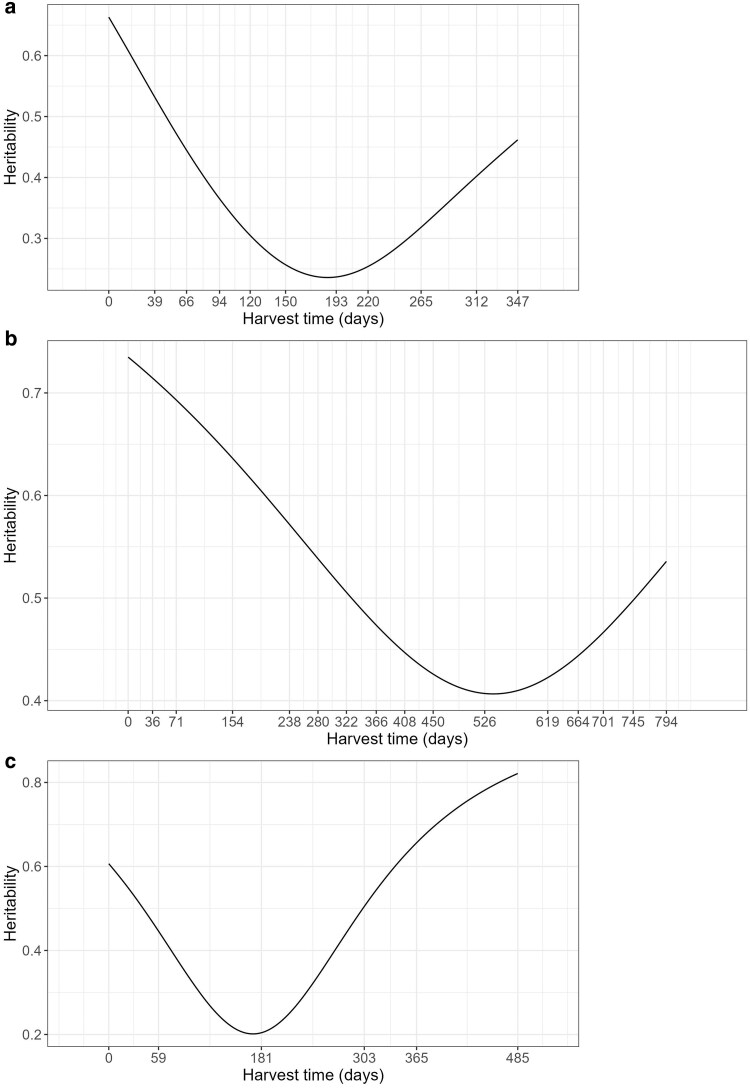
Heritability trajectory over harvests time (days) for a) alfalfa trial (T1); b) *U. brizanta trial* (T5); c) *U. decumbens* trial (T7).

One advantage of RRM is the heritability estimation in function of time, as well as pointed out above by the negative estimate of $\rho_{g_{0}g_{1}}$ the heritability tended to decrease over time as the genetic variance also decreased. The lower heritability estimates (*H^2^* < 0.30) occurred between 120 and 220 days after the first harvest (Fig. 3a). It is noteworthy that the harvest with lower heritability occurred during the late summer to late fall period when alfalfa genotypes are exposed to various stress factors such as high temperatures, cloudy days, and high humidity, along with biotic stress mainly caused by fungal diseases (Fig. 3a).

**Fig. 4.**
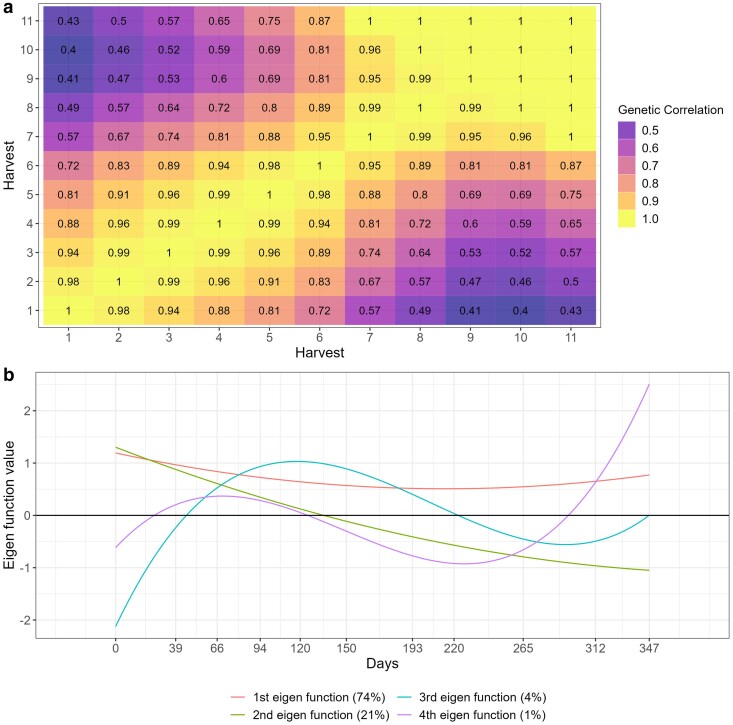
Heat map a) and eigenfunction smooth curves b) illustrating genetic correlations between alfalfa harvests (T1).

The genetic correlation varied from 0.43 to 1.00 (Fig. 4a). The genetic correlation between harvests followed an autoregressive pattern, where harvest closer to each other tends to have higher genetic correlation and those harvests far apart from each other had lower genetic correlation (Fig. 4a). The eigenfunctions can be used in RRM to infer about gene expression over time (Fig. 4b). Where the first eigenfunction had a nearly constant behavior and explained 74% of the genetic variation, this variation represents a common gene poll that is being expressed over time, and explains the simple G × H interaction, since non differential expression was observed (Fig. 4b). The second eigenfunction represents another gene pool, in which explained 21% of the genetic variation that shows differences in gene expression under different environment conditions, explaining most of the complex G × H interaction (Fig. 4b). The third and fourth eigenfunctions explained only 4 and 1% of the genetic variation, also representing complex G × H interactions, where differences on gene expression can be observed over time (Fig. 4b).

#### Genotypes' adaptability, stability, and yield trajectory over time

**Fig. 5.**
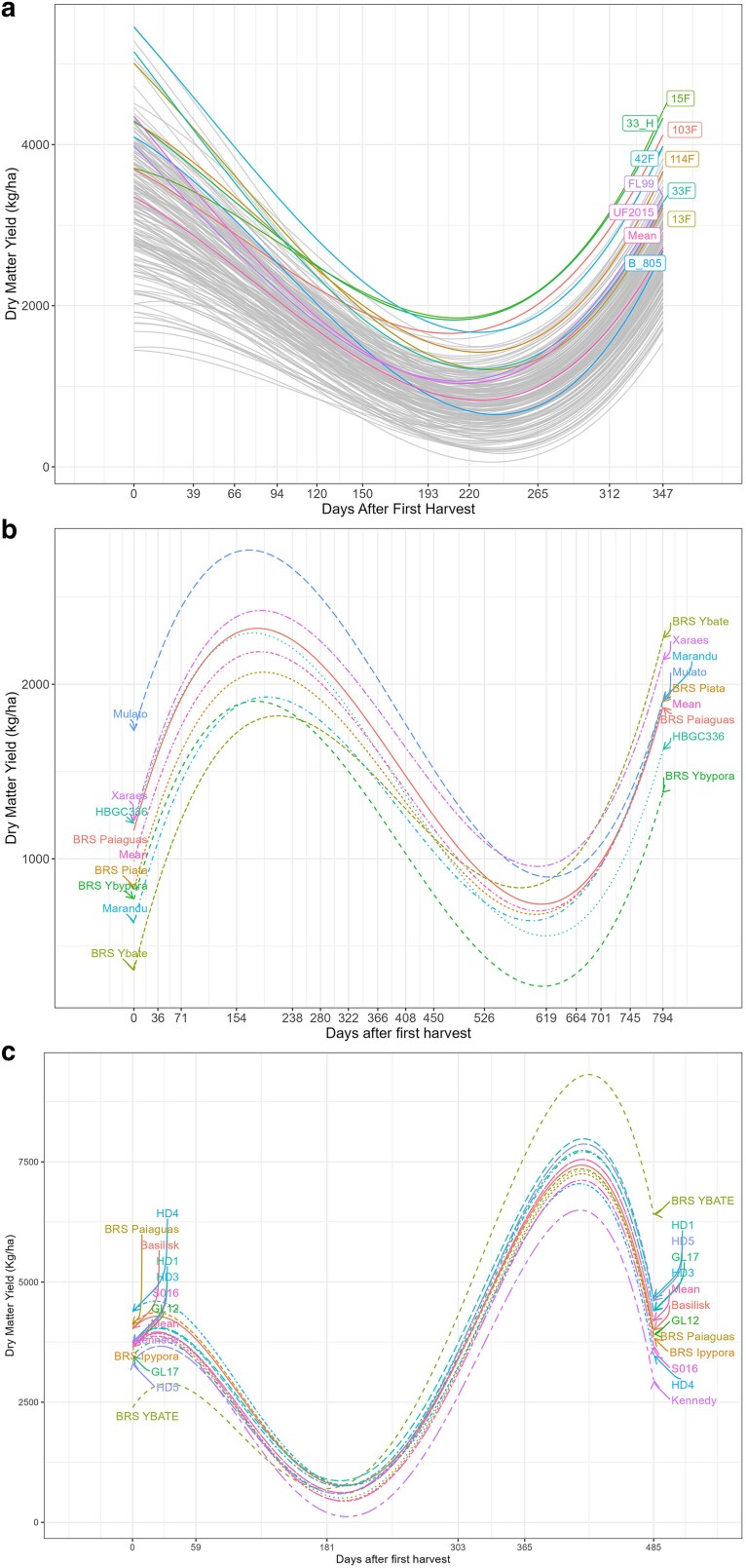
Genotypes' dry matter yield (kg.ha^−1^) trajectory over harvest time for T1—alfalfa trial a); T5—*U. brizanta* b); *U. decumbens* c). The highlighted genotypes represents the better genotypes based on the area under the curve.

Forage breeders are interest in the genotype's behavior over time, a good genotype should have higher yield over time. Therefore, RRM can be a useful tool where genotypes' reaction norms can be plotted (Fig. 5a). There was great variability on the genotypes' reaction norm, and the main changes on ranking occurred between harvests performed between 39 and 220 days. In Fig. 5a, we highlighted seven genotypes that presented higher area under the curve (*A*), i.e. higher broad adaptability, the trial's checks (UF2015, FL99 and B_805), and the mean yield trajectory curve. It can be inferred from Fig. 5a that genotypes 15F, 103F, 33_H, and 42F exhibited superior performance during the stress period (from day 150 to day 265) and can therefore be considered as more tolerant genotypes. All seven genotypes with higher *A* performed better than the checks for most of the harvest evaluated (Fig. 5a).

**Fig. 6.**
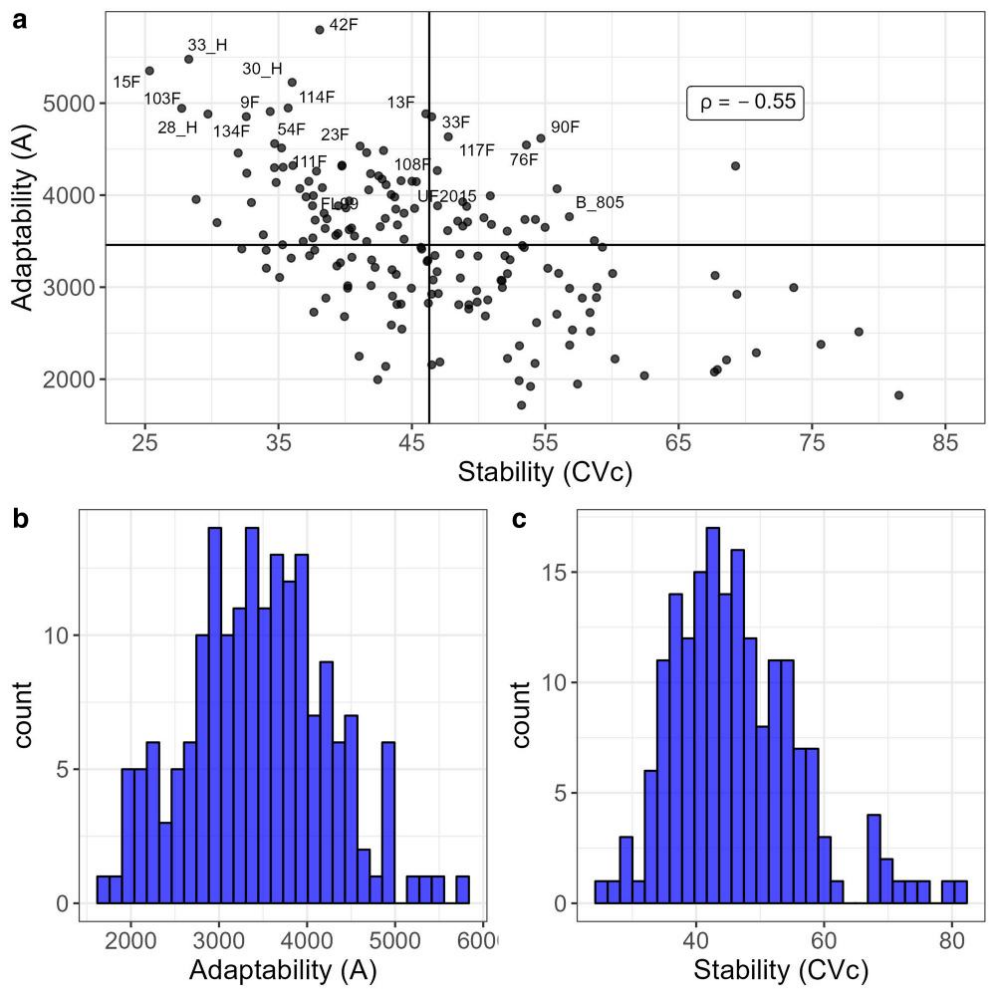
Scatter plot of stability (CV*_c_*) vs adaptability a) with solid black lines indicating mean values of *A* and CV*_c_* for the alfalfa trial (T1); histogram of adaptability b); histogram of stability c).

Genotype selection based only on RRM curves is not feasible when large breeding populations are evaluated and complex G × H interactions are significant. To overcome this difficulty, we proposed the genotype selection based on the area under the curve (*A*) and in the curve coefficient of variation (*CV_c_*), which represents genotypes' adaptability and stability for DMY, respectively. It was observed high variability for *A* and *CV_c_* between genotypes (Fig. 6a and 6b), and the correlation between the two parameters was of median magnitude (−0.55, Fig. 6a); thus, it is possible to select genotypes with higher *A* and lower *CV_c_*, i.e. genotypes presenting high adaptability and stability. For selection purposes, we did a scatter plot showing the genotype's *A* and *CVc* values, where the genotypes to be selected are on the superior left quadrant of the graph (Fig. 6a). The 10% genotypes with higher *A* are highlighted in Fig. 6a.

#### *Urochola brizanta* advanced breeding trial—T5

In this trial, 9 genotypes were evaluated for DMY across 16 harvests. The degree of Legendre polynomial fitted for this trial was three for fixed and one for random part of the model (Table 2). Seven parameters were estimated for RRM.

#### Variance components, heritability, and genetic behavior

For this trial, only the intercept and the first-order polynomial coefficient were needed to model the G × H interaction. The intercept (*g_0_*) and slope (*g_1_*) explained 69 and 31% of the genetic variance (Table 4), respectively. The negative correlation ($\rho_{g_{0}g_{1}}=-0.41$) between *g_1_* and *g_0_* indicated that genetic variances decreased over time. The highest heritability estimates occurred between harvests performed between 0 and 71 days (*H^2^* > 0.70), and the lowest estimates between 450 and 619 days (*H^2^* < 0.43) (Fig. 3b). The first drought season (71–238 days—May to October/2009) had heritability estimates varying from 0.57 to 0.69, whereas the second drought season (450–619—May to October/2010) presented the lower heritabilities varying from 0.40 to 0.43 (Fig. 3b).

**Table 4.** Summary of genetic and nongenetic parameters estimated by RRM for trial T5.

	$g_{0}$	$g_{1}$
$g_{0}$	117,153	−0.41
$g_{1}$	—	53,359
Importance (%)	69	31
${\bar{\sigma }}_{e}^{2}$	282,442	

$g_{0}$ and $g_{1}$ are the random polynomial intercept and the first-order coefficient, respectively. The diagonal elements of the table are the variance components associated with the polynomial intercept and coefficient; and the off diagonal is the correlation between intercept and regression coefficient ($\rho_{g_{0}g_{1}}$). ${\bar{\sigma }}_{e}^{2}$ is the mean error variance through harvests.

**Fig. 7.**
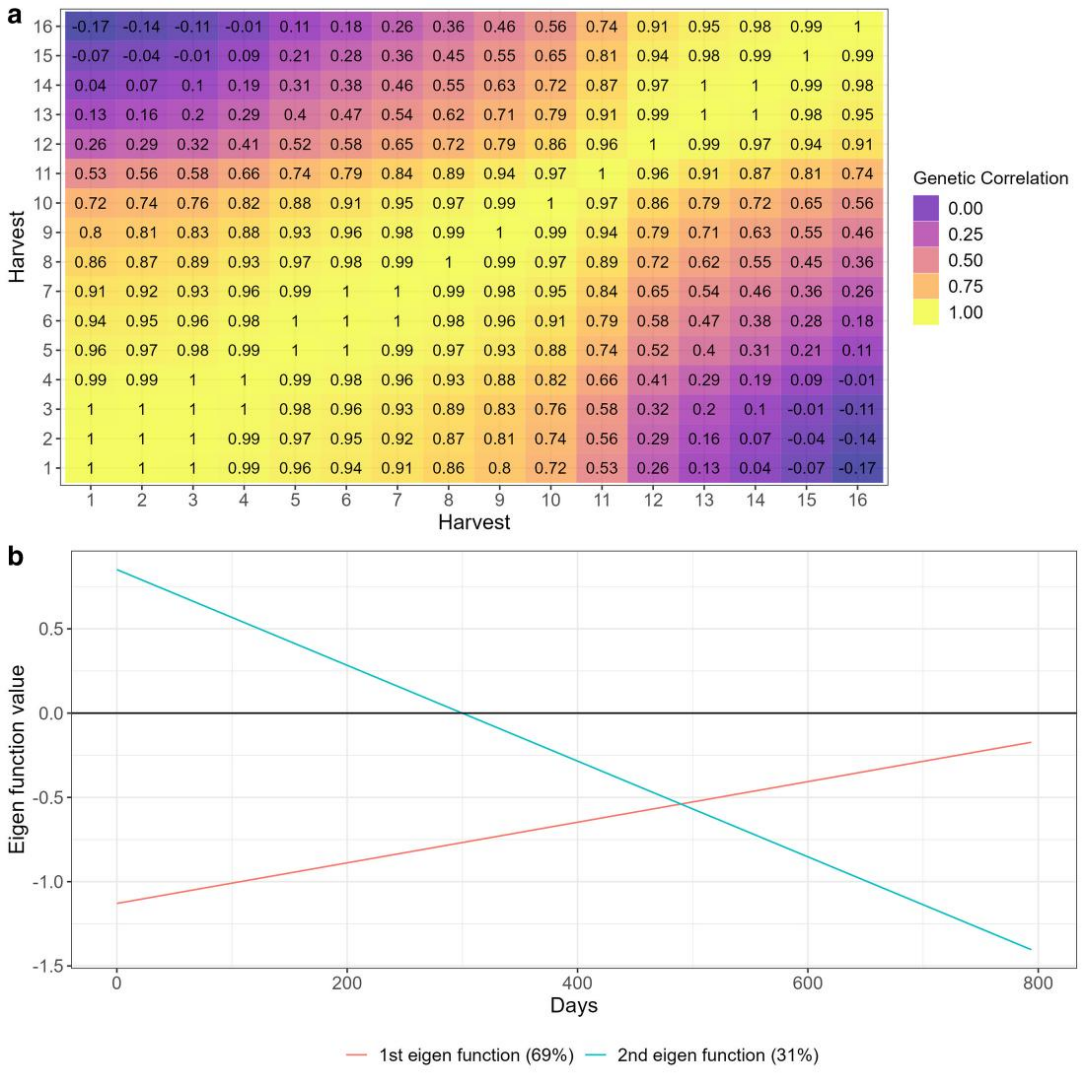
Heat map a) and eigenfunction smooth curves b) illustrating genetic correlations between *U. brizanta* harvests (T5).

The genetic correlation across harvests varied from −0.17 to 1.00 (Fig. 5a and 5b). Like T1 (Fig. 4a), the genetic correlations on T5 followed an autoregressive structure (Fig. 7a), but there was no common factor explaining the G × H interaction for all harvests as negative genetic correlations occurred in this trial (Fig. 7a). This fact can also be explained by the eigenfunctions, where the two eigenfunctions varied over time (Fig. 7b). The first and second eigenfunctions explained the complex G × H interaction, where the gene expression varied over time for the two eigenfunction (Fig. 7b).

#### Genotypes' adaptability, stability, and yield trajectory over time

**Fig. 8.**
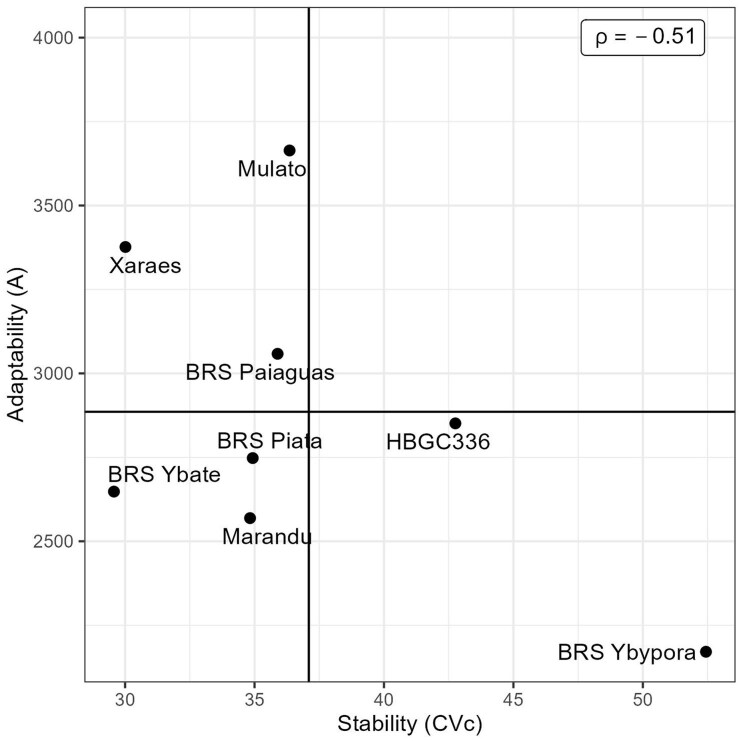
Scatter plot depicting the relationship between genotypes' stability (measured by coefficient of variation—CV*_c_*) and adaptability (measured by area under the curve—*A*).

The genotypes' reaction norms (Fig. 5b) showed that most changes in genotypes raking occurred between days 238 and 664. The maximum DMY was reached between the first drought season (154 days) and beginning of the second rainy season (238 days) (Fig. 5b). This is an atypical behavior and can be explained by the time interval between harvests, reflecting on a longer period of dry matter accumulation (Fig. 5b). Another factor is the number of harvests realized before this period where only three harvests were done until the first drought season (Fig. 5b). Furthermore, atypical climate conditions could happen in this season. As expected, the lowest DMY occurred at the end of the second drought season (619 days) (Fig. 5b). By the atypical behavior of the genotypes on first drought season, the selection of tolerant genotypes to this condition should be done by looking at genotypic values in the period from 450 to 619 days (Fig. 5b). Under drought conditions the genotypes BRS Ybapé, Xaraés and Mulato had the greatest DMY (Fig. 5b). However, BRS YBATE had the lowest DMY at the first harvest, perhaps due to a poor establishment (Fig. 5b). By the reaction norms, the genotype Mulato had the best performance across harvests with good establishment as well as good performance under the drought season (Fig. 5b). The correlation between adaptability and stability was −0.54, indicating that selection can be done for both parameters (Fig. 8). Three genotypes can be selected by presenting higher stability and adaptability (Mulato, Xaraés, and BRS paiaguás, Fig. 8).

#### *Urochola decumbens* advanced breeding trial—T7

In this trial, 12 genotypes were evaluated for DMY across six harvests. The degree of Legendre polynomial fitted for this trial was four for fixed and one for random part of the model (Table 2).

#### Variance components, heritability, and genetic behavior

The intercept explained a small amount (35%) of genetic variance than the first order polynomial coefficient in which explained 65% of the genetic variance (Table 5). Furthermore, the correlation between slope and intercept was positive, indicating that genetic variance increased over time (Table 5). The heritability estimates varied from 0.20 at the third harvest (drought season—August/2018) to 0.82 at the last harvest (beginning of drought season—June/2019) (Fig. 3c).

**Table 5.** Summary of genetic and nongenetic parameters estimated by RRM for trial T7.

	$g_{0}$	$g_{1}$
$g_{0}$	165,648	0.68
$g_{1}$	—	299,828
Importance (%)	35	65
${\bar{\sigma }}_{e}^{2}$	694,316	

$g_{0}$ and $g_{1}$ are the random polynomial intercept and the first-order coefficient, respectively. The diagonal elements of the table are the variance components associated with the polynomial intercept and coefficient; and the off diagonal is the correlation between intercept and regression coefficient ($\rho_{g_{0}g_{1}}$). ${\bar{\sigma }}_{e}^{2}$ is the mean error variance through harvests.

**Fig. 9.**
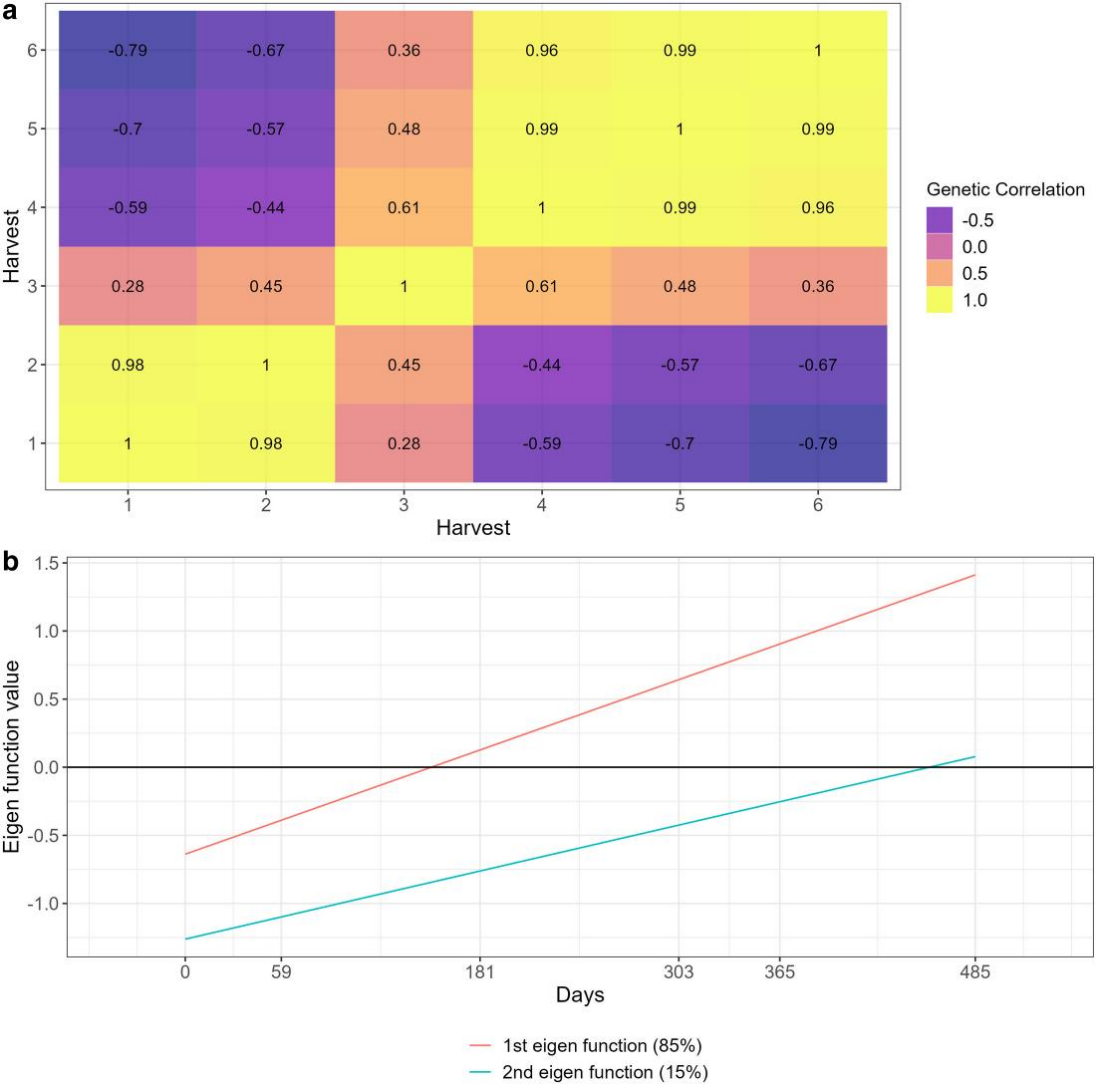
Heat map a) and eigenfunction smooth curves b) illustrating genetic correlations between *U. brizanta* harvests (T7).

The genetic correlations across harvests varied from 0.99 to −0.79, indicating a high and complex G × H interaction effects (Fig. 9a). As it happen for T1 and T5, the RRM approximated the (co)variance structure into an autoregressive structure on T7 (Fig. 9a). The two estimated eigenfunctions varied across time, indicating there was not a common gene pool expressing in the same way for all harvests evaluated (Fig. 9b). Both gene pools represented by the eigenfunctions are expressing differentially across harvest, explaining the strong complex G × H interaction occurred for this trial (Fig. 9b).

#### Genotypes' adaptability, stability, and yield trajectory over time

**Fig. 10.**
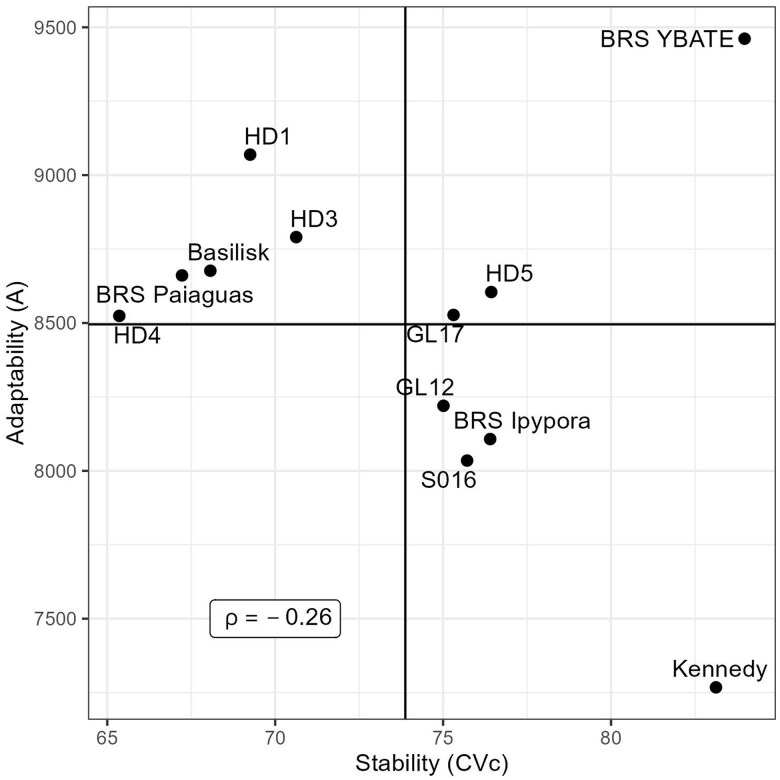
Scatter plot depicting the relationship between genotypes' stability (measured by coefficient of variation—CV*_c_*) and adaptability (measured by area under the curve—*A*).

The most changes in genotypes raking occurred after the first drought season at harvest performed on day 181 (Fig. 5c). The highest DMY occurred at day 365 (rainy season), and the lowest DMY occurred at the first drought season (day 181) (Fig. 5c). The genotype BRS YBATÉ, also evaluated in trial T5, had the same behavior, where it had a poor establishment (day 0–59) and a good recovery after the first drought season, being the best genotype in all harvests after the drought season (Fig. 5c). The correlation between *CV_c_* and *A* was −0.25, indicating the possibility of selecting adaptable and stable genotypes (Fig. 10). Although the genotype BRS YBATÉ had the best adaptability, it was one of the most unstable genotypes with greater variation in DMY across harvests (Fig. 10). Five genotypes were identified having good stability and adaptability (HD1, HD3, Basilisk, Paiaguás and HD4) (Fig. 10).

## Discussion

Our study focused on the analysis of longitudinal DMY data from ten different trials and four perennial forage species, utilizing RRM methodology. Our approach included the estimation of variance components, and the selection of genotypes based on their adaptability and stability for DMY. Through this analysis, we aim to gain deeper insights into the performance of different genotypes over time, and to identify the most suitable candidates for future breeding programs.

### Goodness-of-fit evaluation

Proper modeling of genetic effects over time is crucial for accurate analysis of yield data in forage perennial species (Faveri et al. 2015). RRM has proven to be an effective method for dealing with longitudinal records in animal breeding (Shaeffer 2004; Kranis et al. 2007) and can be adapted for use in analyzing longitudinal data in plants. As observed in all trials presented in this study, DMY records were obtained at unequally spaced intervals due to the seasonality inherent in forage growth under tropical and subtropical climates (Reis and Rosa 2001). Infinite-dimensional methods, such as RRM, offer an advantage in this context, as they can effectively handle unequally spaced records. This is because yield trajectories are continuous functions of time, meaning that an infinite rather than finite number of measurements is required to fully describe a trait in an individual (Kirkpatrick and Heckman 1989; Kirkpatrick et al. 1990).

The selection of the random polynomial order in RRM can be achieved by using goodness of fit and parsimony criteria, as demonstrated by Corrales et al. (2015). In this study, we adopted the BIC approach, as suggested by Rocha et al. (2018), to select the random polynomial order. However, choosing an appropriate fixed function to model the phenotypic curve shape can be challenging. In cases where the overall trajectory is linear, fitting the fixed part of the model using a function can be straightforward. However, due to fluctuations in forage DMY caused by changing climate conditions over time, the trajectory will inevitably follow a nonlinear pattern. In such cases, the fixed part of the model can be treated as factor variables, which require more degrees of freedom (Shaeffer 2004). To address this issue, we proposed using a smooth loess function as the fixed function and selecting the polynomial order graphically (Fig. 1). By using a mathematical function instead of factors, we were able to obtain smooth trajectories over time regardless of the number of observations (Fig. 5).

### Exploring genotype by harvest interaction: reaction norms, genetic correlations, and autoregressive patterns

In forage breeding trials, the G × H interaction is a crucial aspect that needs to be understood to identify the best performing genotypes under different environmental conditions and at different stages of growth. This interaction can be studied using various statistical approaches, such as reaction norms, genetic correlations estimated by covariance functions and/or eigenfunctions across time (Filho et al. 2023).

Reaction norms are graphical representations of the relationship between genotypes and the environment. When interpretation is done by reaction norms (Fig. 5), it is essential to look for deviations from parallelism, such as intersections, divergences, or convergences (Van Eeuwijk et al. 2016). Divergence and convergence occur when simple G × H is acting over time, meaning that the genetic variance is increasing (diverging) or decreasing (converging) (Crossa et al. 2004). The complex G × H interaction occur when the reaction norms intersect, meaning that is a lack of genetic correlation between measurements (Elias et al. 2016; Van Eeuwijk et al. 2016). In all trials analyzed, the estimated genetic correlations tended to follow an autoregressive pattern, where harvests closer to each other had higher correlations, whereas harvests far apart had lower genetic correlation (Figs. 4a, 7a and 9a). This autoregressive pattern is very common in longitudinal data in perennial species (Apiolaza and Garrick 2001; Yang et al. 2006; Giri et al. 2019; Peixoto et al. 2020; Bornhofen et al. 2022; Murad Leite Andrade et al. 2022) and have a satisfactory biological explanation, indicating that genes are expressing differently according to the environmental conditions and genotypes' age (Falconer and Mackay 1996; Gauch and Zobel 1996).

Another way to interpreting the G × H interaction in RRM is through eigenfunctions. Eigenfunctions are analogous to eigenvectors (principal components). Each eigenfunction is a continuous function that represents a possible evolutionary deformation of the mean yield trajectory (Kirkpatrick et al. 1990). When the eigenfunction is nearly constant, it means that the eigenfunction captured a gene pool that was equally expressed over time (Kirkpatrick et al. 1990; Rocha et al. 2018; Peixoto et al. 2020). On trial T1, the first eigenfunction had a constant behavior and it is explaining the general adaptability gene pool equally expressed over time and the positive genetic correlation (Fig. 4b). The other eigenfunctions from trial T1 are explaining the lack of genetic correlation, where the gene pools had differential expression over time (Fig. 4b). For trials T5 and T7, there was negative genetic correlation over time indicating that there was not a common gene pool expressing equally through the harvests, therefore no eigenfunctions had a constant behavior (Figs. 7b and 9b). As demonstrated in this study, eigenfunctions can explain the G × H interaction as introduced by Falconer and Mackay (1996), where genotype by environment interaction can be considered a pleiotropic effect of a trait evaluated across environments.

### Genotypes' reaction norm, adaptability, and stability

A reaction norm defines a genotype-specific function that translates environmental inputs into a phenotype (Van Eeuwijk et al. 2016). The differential genotypes' response to the environment (genotype plasticity) generates the genotype by environment interaction. In this study, it was observed a higher variation for genotypes' reaction norms for all trials interpreted (Fig. 5), indicating strong complex G × H interaction. Reaction norms are very informative for analyzing perennial forage over time, it allowed us to observe the behavior of each genotype, identify periods where seasonality occurred, and identify those genotypes which respond better to environmental stress. However, interpreting reaction norms for each genotype can be difficult in large breeding populations. Therefore, breeders usually use specific-genotype parameters, such as intercepts, slopes, curvatures, and variances.

These specific-genotype parameters are called sensitivity, adaptability, and stability parameters in plant breeding literature and they facilitate the modeling of complex genotype by environment interactions (Finlay and Wilkinson 1963; Eberhart and Russell 1966; Lin and Binns 1988). Another way to select genotypes based on adaptability and stability is computing an index regarding the predicted genotypic values across all environments (Kelly et al. 2007; Faveri et al. 2015; Rocha et al. 2018). In this study, we proposed an adaptability measure the genotype's area under the curve, which has a closer meaning to the total DMY across all harvest (*A*) and overall genotype's performance. For stability parameter, we proposed the use of the curve coefficient of variation (CV*_c_*). Stability can also be referred as risk in variety adoption, where the most stable genotype should have lower variance across environments, meaning that the genotype is more predictable (Eberhart and Russell 1966; Lin and Binns 1988). The coefficient of variation is a broad used and easy to interpret parameter in different disciplines. In time series, mainly in economics it is frequently used to infer about the risk and uncertainty in shares on the stock market exchange (Curto and Pinto 2009). Therefore, we used this concept to infer about DMY stability in genotype selection, in which genotypes having lower CV*_c_* will have higher stability and will also have lower variation across harvests.

## Conclusion

This study demonstrated the effective application of RRM for analyzing longitudinal data in various forage breeding trials. Our findings highlighted the importance of RRM in identifying G × H interactions and estimating adaptability and stability, using measures such as the area under the reaction norm curve and the curve coefficient of variation. By utilizing RRM in longitudinal datasets, we were able to better understand genotype seasonality through predicted reaction norms. Based on these results, we recommend the use of RRM for analyzing longitudinal traits in forage breeding trials, as it provides valuable insights and enhances our ability to interpret and evaluate genetic performance over time.

## Data Availability

The phenotypic data and R code used in this study are available at github (https://github.com/claudiocff/RRM-and-FAMM-asreml-two-step).
